# Research models for financing social business: theory and practice

**DOI:** 10.1016/j.heliyon.2019.e01599

**Published:** 2019-05-18

**Authors:** Nurkhodzha Akbulaev, Yusif Aliyev, Turan Ahmadov

**Affiliations:** Azerbaijan State University of Economics (UNEC), Faculty of Turkish World Economics, Department of Economics and Business Administration, Baku, Azerbaijan

**Keywords:** Economics, Business

## Abstract

In the establishment of social enterprises with further functioning, there exists the problem of accessing investments. Therefore, theoretical and managerial aspects of the models formation of financing social entrepreneurship in the global economy are discussed in the article. The purpose of this research is consideration in financing methods of social enterprises for competitiveness in provision of the highest quality services and improvement of living standards. In order to achieve this purpose, methods of logical analysis, expert assessments, comparison method as well as systems analysis, synthesis, induction, deduction and analogies were applied. Moreover, the literature review on problems of social entrepreneurship development were analyzed. The approaches of social entrepreneurship definition and its disparity from charitable organizations and traditional businesses were highlighted. Furthermore, comparative analysis of the European and American models of financing social business was provided. The results are presented in five tables. The discussion of financial support for social enterprises is more active at all levels of government compared with the United States. In conclusion, the investment transparency of such enterprises with open accounting system should be driven by future development of social entrepreneurship. In view of this, conditions for the formation of social financial market can be created.

## 1 Introduction

Recently, significant changes in the world economic system brought about the realization of human resources as a key player among the factors of production. Meanwhile, the investment in human capital is more effective than other factors. The strategic vision and the main trend of sustainable and dynamic socio-economic progress of countries form socially oriented development with social innovation as one of the basic sources. Innovative technologies include extraordinary ways of solving social problems without any analogues. Thus, the emerging companies around the world focus on social problems, so-called “social enterprises”.

The social entrepreneurship gets special significance in periods of the economic and financial crises. First of all, with the increasing social challenges at this time, finding a solution would be beneficial to the society. Secondly, social entrepreneurship contributes to the creation of a favorable business environment, active citizenship support, innovation and public solidarity, as well as conditions for more flexible and timeliercrisis recovery.

A very crucial problem for social enterprises in the process of establishment and further functioning is access to investment. The development of instruments for financing social enterprises will enable them become competitive in providing the highest quality services, leading to improvement in the standard of living.

## 2 Theory

### 2.1 The theory of social entrepreneurship

The present article is based on general scientific and special methods, with consideration of processes and phenomena in the interconnection. The theoretical aspect of the study is based on fundamental principles of the economic theory, as well as scientific works of domestic and foreign scientists on the problems of social entrepreneurship. In disclosing foreign experience for social entrepreneurship development, methods of logical analysis, expert assessments, comparison method as well as systems analysis, synthesis, induction, deduction and analogies methods were applied.

The world scientific society and specialists as representation of modern business have recently begun a study to focus on the problems of social entrepreneurship development at the national and global levels. Various social aspects of entrepreneurship were highlighted in the works of many scholars such as Zvereva (2015), Safarov (2014), Popov et al. (2017), Moskovskaya (2011), Semenova (2017) etc. In foreign scientific works, the problems of social entrepreneurship development were investigated in the works of some specialists, such as V. Weiner, A. Carroll, M. Van Marreviyk, J. Sandal, F. Sprekli, Acs et al. (2015), Acs et al. (2018), Alemanno and Cottakis (2017), Bedford and Carayannis (2018), Dey and Steyaert (2018), Tan (2014), Wexler (2006), Wexler (2018) etc. However, in these studies, very little attention is given to the generalization of modern experience in formation and functioning of effective models in financing social entrepreneurship as important scientific rationale for solving the acute domestic problems. Therefore, the purpose of the present article is to compile the modern experience of models in financing social entrepreneurship and ways of practical implementation in different countries of the world for recommendations in improving fund domestic model, as well as the development and functioning of social entrepreneurship.

Prior to the study of the organization specificity under social business financing, the main conceptual aspects of the topic and the concept of social entrepreneurship must be considered.

Our findings suggest that complexity in the definition of “social entrepreneurship” concept is derived from multidimensionality of the entrepreneurship with reflection on a wide range of tasks and existing features. Under the headline, the priority for this type of business is not profitability, but providing solution or mitigation to existing social problems. The essence of social entrepreneurship can be better understood by reading the three main approaches to its definition.

Thus, in the countries of South and North America, social entrepreneurship is considered as the entrepreneurial activity of non-profit organizations with the income for realization of the organization's statutory goals. The objective of statutory goals for non-profit organizations focuses on social problems, services to the target group, for which the organization was established with improvement in the quality of life (Borzaga, 2018).

Further approach to the definition of social entrepreneurship is related to the fact that its social effect on the entrepreneurial activity is more important than the financial efficiency unlike in simple business. Here, the subject of social entrepreneurship is business enterprises with pronounced social purpose.

Another approach is used by most international private and public foundations, created for developing and supporting this sphere of social and economic activity. The Schwab Foundation for Social Entrepreneurship (Switzerland), the Skoll Foundation (USA) and the Ashoka Foundation (India) (Matthews and Liguori, 2018, p357) defined social entrepreneurship as an innovative entrepreneurial activity which causes social transformations in the society and communities.

The contrast to this approach shows social entrepreneur as the leader. The activity of these organizations is to search for social innovators around the world, create favorable conditions for work, support and acknowledgment. The subject of social entrepreneurship is the person and the activity. Here, the form of organizing own business is not significant and it may differ from the initiative group in the community as obtainable in private business or research institutes.

## 3 Materials and methods

In the course of the study, general scientific research methods were used such as methods of deduction and induction, comparative analysis, synthesis, as well as method of scientific review of the source base.

## 4 Results

Based on this, social entrepreneurship can be defined as a business system, the components of which are social enterprises. The activities of such social companies focus on making profit while solving social problems.

The formal definition of social entrepreneurship was given in 2002 by the Department of Commerce and Industry of the British Government. They defined social entrepreneurship as a business performed primarily to achieve social goals rather than to generate profit for the owners and shareholders. The incomes are mainly directed towards development of business or public affairs (Bataeva, 2015 p 221–223).

The Finnish Ministry of Labor proposed the following: above all, social enterprise is business, like any other enterprise, with the goal of making profit by supplying goods and services to the market. However, what makes it different from others is that at least 30% of the company's employees must be people with disabilities (disabled). The company can include the unemployed and people with disabilities for a long time, by providing jobs for them. The social enterprise helps to create a more balanced, humane society, that is, one in which every person can participate (Bataeva, 2015 p 221–223).

For accurate characteristics of social enterprise essence, we will consider the criteria that most closely correspond to social entrepreneurship:

– Social influence (focus on solving or mitigating a specific social problem). The social effect is result oriented, not a by-product of the activity (it should not be identified with the social responsibility of enterprises). Social goals should be determined in the Chapter of the enterprise or in other constituent documents, accepted by the founders with some mandatory goals;
– Innovativeness (application of new approaches and new ways of solving old and new social problems);
– Profitability and financial sustainability (independence from external financing). The main task of enterprise is profitability regardless of traditional or innovative aspect of the company. The main goal of social enterprise is independent earning (excluding funds from sponsors) and independent spending, earned on the provision of solution to social goals. Profit is not to be distributed for private purposes;
– Democratic management. Furthermore, this criterion confirms the difference between social enterprise and socially responsible analogue. All stakeholders are involved in the decision-making process, which illustrates the openness and transparency of the social enterprise. Democratic management is easily applied in social enterprises, created by public organizations, with the general meeting being the highest governing body. Moreover, cooperative can provide democratic management, since the scheme is 1 shareholder equals 1 vote;
– Income reinvestment for business or for social purposes. We highlight the following approaches for profit reinvestment:
1) All profits are reinvested into the expansion. Such approach is inherent in social enterprises created by people with special needs or socially vulnerable categories of people targeted for employment;
2) Apart of the profit is reinvested, while a part is spent on social goals. Such distribution is characteristic of social enterprises, created by public organizations or charitable foundations. The assigned part on social purposes is paid by the public organization. This scheme is the most common in the world, since it contributes to the development of the commercial component and create better social effect;
3) All profits are channeled towards achieving the social effect. This approach is mainly used in tandem (public organization and private enterprise). Public organization leases some means of production for commercial activity to the private enterprise, and all profits are returned to the public organization. This approach for profits reinvesting is the least common.

In summary, we define social enterprise as a legally registered organization whose activity is not centered on profit, but it is targeted at solving social and environmental problems. The profit is directed mainly to the development of the organization or to public affairs.

While recognizing the important role of such phenomena in society such as charity or corporate social responsibility, we noted the specific features of social entrepreneurship and its dissimilarity with other phenomena, and also what unites them. Social entrepreneurship is at the junction of traditional entrepreneurship and charity. Social entrepreneurship takes social orientation from charity, and business goes from the entrepreneurial approach (Table 1).

**Table 1:** The differences between social enterprise, charitable organization and traditional business.

The social enterprise	The charity organization	The traditional business
- Socially oriented structure; - does not depend on external sources of financing; - only starting capital is required; - receives income from its own activities, the purpose of which is mitigating or solving specific social problems; - profit is reinvested.	- Socially oriented structure; - depends on external sources of funding, donors; - receives grants and donations that are sent for solving social problems; - no profit.	- Commercial structure; - does not depend on external sources of financing; - only starting capital is required; - receives income from its own activities, the purpose of which is to maximize profits; - profit is distributed among shareholders.

Thus, social entrepreneurship is symbiosis of charity and business approaches in social problems solving. Unlike the charitable organizations which have a non-profit motive, social business generates income. In contrast to traditional businesses that work for the sake of profit, social entrepreneurship performs social functions and works where the state cannot (due to lack of funding). The advantage of social entrepreneurship is providing solutions to social problems without government intervention.

In addition, social entrepreneurship does not need to be identified with social responsibility, whereby certain business structure adheres to policy that takes into account the socio-economic and environmental interests of the community or territory where it operates. At the same time, a socially responsible business can contribute to the development of social enterprises. For instance, representatives of corporations can provide advisory support to those who control social enterprises (Borzaga, 2018). These two types of enterprises are equally important, but in contrast to socially responsible business, social enterprise is not aimed at increasing the profit of shareholders.

The company needs an initial financial assistance or support and social enterprise is no exception. In most cases, it is necessary to fund throughout operations, since the main goal is to create social value.

Safarov S.M. noted that social enterprise is business of the future. The fact of it not being profit oriented makes the entrepreneur get more from the activity, for example, creating social value (Blank, 2018 p 30–32). Therefore, a good number of the international organizations and foundations are interested in creating social enterprises as the newest stage in the functioning of entrepreneurship.

The development and financing of social entrepreneurship is carried out by some international organizations and foundations such as: Western NIS Enterprise Fund (USA), Skoll Foundation (USA), The Social Enterprise Fund (Canada), UnLtd (United Kingdom), Ashoka (India), Renaissance (Ukraine), British Council (UK) and others. Among them, there are organizations in which the support of social entrepreneurship is one of the activities (Western NIS Enterprise Fund, Renaissance, British Council) and those, for which the development and spread of this entrepreneurship, is the main goal of the activity (Skoll Foundation, The Social Enterprise Fund, UnLtd, Ashoka) (Bataeva, 2015 p 221–223).

The assistance of international funds and organizations differ from domestic financing of social enterprises by quite wider support, namely: the irrevocable provision of funds (grants), the provision of interest-free or preferential loans, methodological assistance, mentoring and the like. A large number of social enterprises participate in contests to receive a grant, and not interest-free loan. In our opinion, the latter is more efficient, since it stimulates social enterprise to developing and generating profit for repaying a loan. At the same time, grant financing has a number of advantages, such as: irrevocable receipt of funds, low cost of raising funds with no further obligations on organization functioning. Disadvantages are as follow: targeted use of funds, periodic accounting and high competition in receiving a grant.

Lending is an important tool for financial support of social enterprises. It is impractical to put traditional business and social entrepreneurship in the same lending conditions as common functional structure, since the purpose of activity is different.

Thus, the specificity of social business has no tendency of achieving profit by the initiators. Resolving acute social problem or performing significant social task is much more important to them. But the approach to the solution (social task) contains all the elements of business such as: management, resources, procurement, personnel, finance, accounting, promotion and advertising.

The differences in the focus of activities lead to differences in the structure of sources in social enterprises financing. Moreover, the development of a system, designated for financing social entrepreneurship, has own characteristics in some areas. Hence, there are two main models of financing social enterprises, namely: the European and the American.

In the American model of social enterprises financing, the main source of finance is profit from the activities of social enterprise. Meanwhile, the external sources of funding are additional. The American model of social entrepreneurship financing does not provide special arrangements for the government's funding of social enterprises and the creation of special government authorities with competence to support. The functions of social enterprises assistance in the United States are assigned to the extensive network of existing government authorities of the Small Business Administration. Also, the financial support for social enterprises is provided by the Department of Social Innovations, with an annual contribution of about USD $ 2 million for the promotion of advanced innovative projects, including social enterprises (Borzaga, 2018). Various charitable organizations, venture capitalists, “business angels”, investors of influence and others are central in enhancing the development of social entrepreneurship. Accordingly, the American model of social entrepreneurship financing is more “economical” in terms of government budgets and more market-oriented.

In the European model of social entrepreneurship financing, the share of profits in the structure for sources of the same funding is irrelevant. The key point here is on solving social problems.

In contrast, there are conceptual differences in the system of state support for the European and American models. A special legal framework was created in European countries (the Law on Social Cooperatives (Italy, 1991), Law 4019/30-9-2011 “On the Social Economy and Social Enterprises” (Greece, 2011), The Public Services (Social Value), Act (State Services (Social Values), Act (Great Britain, 2012) to regulate the processes of formation, functioning and government support. However, in the USA, legal aspects of regulation and government support for social enterprises are normalized within the framework of legal sphere common to other business entities. In this country, the government's support for social entrepreneurship development focuses on: “the elimination of legal administrative barriers, legislative maintaining of preferential loans, social partnership in government organizations, business and the non-profit sector,” preferences access in licensing and certification of social enterprises in such spheres as social services, education and medicine. The social enterprises are guaranteed the opportunity to receive social orders from government agencies (Diamond, 2015 p 141–155).

The subsidiary structures of social enterprises instances are represented as banks, mostly non-bank financial institutions (credit unions). Thus, in Britain, there are many grants for projects in social entrepreneurship. The Big Society Capital Bank, founded in April 2012, is aimed at supporting social enterprises. The total budget of £600 million should be distributed through the intermediaries for the benefit of social enterprises and public organizations. The innovative financing method is the company's shares: each member of the community invests small amount in social enterprise (Alemanno et al., 2017).

Another example is the Austrian AG “ERSTE-Bank” that directs 60% of profits for social purposes. This company provides preferential loans to the best social programs in the British pilot project in order to support social entrepreneurship in Ukraine, Croatia, and Serbia. The assistance can also be provided by regional, local, state-wide associations of social enterprises (the associations of social enterprises in France, Italy), and private foundations (the Klaus Schwab Foundation in Switzerland, with the founder of the forum in Davos), etc.

The specificity of the American method in financing social entrepreneurship development is the availability of extensive and stable system in private support as charitable foundations (Borzaga, 2018). The following are among the largest systems: the Roberts Enterprise Development Fund in sphere of support for the newly created social enterprises; the Goldman Sachs Fund in sphere of social enterprises competition in commercial activities. Gradually, more attention is given to individual social entrepreneurs as part of intensive educational programs and/or grants, including international programs such as: the Draper Richards Foundation, Echoing Green, Skoll Foundation, Ashoka, and Schwab Foundation. Below is the infrastructure of foundations and non-profit organizations that finance social enterprises (Table 2).

**Table 2:** US foundations and non-profit organizations that finance social enterprises.

The name of company	The principles of activity
Social enterprise alliance www.sc-alliancc.org	The Alliance defines itself as “a membership organization whose goal is to mobilize communities of non-profit organizations and donors for the purposes of interaction to implement profit-making strategies”.
ACUMEN fund www.acumcnfund.org	The non-profit international business fund that solves problems of poverty around the world. It directs financial recourses to provide people with water, housing, energy and health services.
Ashoka: innovators for the public www.ashoka.org	The international Association of Leading Social Entrepreneurs. It pays scholarships to laureates, provides professional support by establishing links between like-minded social entrepreneurs.
Craiglist foundation	This Foundation provides professional support to leaders in the non-profit sector and aspiring social entrepreneurs in the form of affordable or free education.
Draper Richards foundation www.drapcrrichards.org	This Foundation provides financial support as a business coach for social entrepreneurs.
Echoing green www.ochoinggrccn.org	The organization finances social entrepreneurs at the first stage of activities and provides other forms of support for translating into action original ideas aimed at achieving social change.
Ewing Marion Kauffman foundation www.kauflman.oig	The Foundation operates in two directions: supporting all types of entrepreneurship and improving the quality of education.
RSF social finance www.rsfsocialfinancc.org	The catalyst for socially oriented projects that promotes positive social change.
Shorcbank corporation www.shorcbankcorp.com	The holding company dedicated to the development of local communities and environmental protection.
Skoll foundation www.skollfoundation.org	The Foundation provides strategic support to individuals with initiation of long-term social changes.
Surdna foundation www.surdna.org	The family foundation that supports, shapes and promotes effective long-term solutions, aimed at achieving social changes.

In summary, we show a table of comparison with reflection on the similarities and differences in the European and American models of financing social entrepreneurship.

A comparison of financing social entrepreneurship in Europe and the USA revealed advantages for both models (Table 3). Particularly, in the countries of Western Europe, businessmen actively apply the practice of involving stakeholders in management with opportunities for government participation in the process of social entrepreneurship development. On the other hand, the United States can be an example for Europe to expand the scope of social enterprises and the types of legal forms of such enterprises, as well as using of government contracts to purchase products.

**Table 3:** Comparison between the American and European models of financing social entrepreneurship.

The criterion of comparison	The American model	The European model
The main source of funding for social enterprises	Foundations (Skoll Foundation; Schwab Foundation for Social Entrepreneurship; William and Flora Hewlett Foundation; David and Lucile Packard Foundation; Aspen Institute) Payment for goods/services Private donations	Payment for goods/services Grants Investor capital Private donations Membership fee
The goals of social business	This is an opportunity for self-financing offered to non-profit organizations.	This is a business with social goals.
The method of profit distribution and its target direction	The method of profit distribution may include enriching the owners of the enterprise	The method of profit distribution may include enriching the owners of the enterprise
The availability of government programs and strategies for financial support of social business	There are practically no government programs and strategies for the development of social enterprises	The active work is being done in this direction

Instead, social enterprise in the United States is largely left to the private and civil society sectors. Moreover, in the United States, more emphasis is generally placed on revenue generation in support of a wide range of social purposes with attracting beneficiaries in the earned income activity and orientation on the disadvantages as well as improved well-being. In this case, the national-level legal form was not created for social enterprise. Under significant state-level changes, profit legal forms allow the existence of social and profit goals (for example, low-profit limited liability corporation, benefit corporation and social purpose corporation).

The difference between Europe and the United States is the internal governance of the social enterprise. In the European context, the governance of the organization carries greater importance due to their involvement in the democratic advancement of the economy. Indeed, the European social enterprise focuses on autonomous development, decision making exclusive of capital ownership and participation of multi-stakeholders in the governance of the organization. All this points to the cooperative roots of social enterprise in Europe. In terms of autonomy, the hallmark of European social enterprises is the establishment and management by citizen groups rather than public or private entities. Though, they can receive significant funding from these sources. As such, public–private partnerships are not included in the conceptualization of social enterprise, even being at times in the American context.

As shown by the international experience, a wide range of methods for social enterprises financing (Table 4) provide favorable conditions for expanding the range of social enterprises.

**Table 4:** The main sources and instruments of financing social enterprises.

The source of financing	The tool of financing
Entrepreneur	Equity Capital (Bootstrapping)
Population	Crowdfunding
Private investors	Accelerators
Private investors – legal entities	Incubators
Charitable and public organizations Business organizations (large) International organizations	Fellowships and pitch competitions Grants
Private investors Charitable and public organizations Business organizations (large) State	Social impact bonds
Private investors	Business Angels (Angel investors)
Private investors Business organizations (large)	Transformative investment (Impact investing) Venture capital
Banks	Loans
Credit unions Microfinance institutions	Microfinance (microcredit)

There are specific financing with the orientation to social enterprises fund (according to foreign practice):

- crowd funding is an open call via the internet to collect financial resources in the form of cash donations, sometimes in exchange for future products, services or rewards;
- social impact bonds (Social impact bonds is a new approach to social programs, aimed at raising funds from charitable organizations and the investors for financing of preventive social programs;
- Impact investing – is focused on making business profit with measurable social results, while solving the social problems of poor and marginalized populations (Khanna, 2018);
- Microfinance (microcredit) is one of the aspects whereby a small amount of money is made available by the bank or other organization. In general, the basic idea of microfinance correlates with the idea of social entrepreneurship and consists in overcoming poverty, thereby ensuring the economic sustainability of socially unprotected segments of the population and supporting integration into the labor market through the development of small enterprises (National Guard and Reserve Entrepreneurship Support Act of 2018, p7).

The EU provides funds to finance social enterprises; for example, in Poland, such enterprises received resources from the European Social Fund (European Social Fund) under the Sectoral Operational Program of Human Resources Development from 2004-2006 (Kiseleva et al., 2017 p 250–259).

**Fig. 1:**
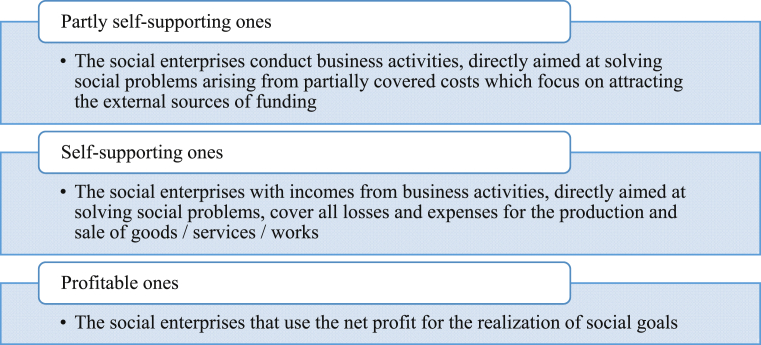
Classification of social enterprises according to the degree of financial soundness.

A study of social enterprises functioning shows that the vast majority is not financially independent with opportunities for raising funds from charitable or business organizations. At the beginning of operation, not every social enterprise can profitably organize economic activity. In most cases, newly created social companies focus on covering losses on the basis of the author's classification of social enterprises by the degree of financial soundness (Fig. 1).

The features of partially self-sustained and self-supporting social enterprises consist of the fact that business activity is directly aimed at solving certain social problems. The differences relate to the ability of partly or fully recovering the costs of operation. In accordance with the type, newly created and non-profit organizations with the goal of being a social enterprise, usually and partly belong to self-supporting social enterprises.

The self-sustained social enterprises are small number of social companies, depending on the subsequent outcome of business can become partly self-supporting or profitable. The profitable social enterprises have effectively organized business activities with not only finance expansion, but also with investment in solving certain social problems.

In contrast with the existing approaches to the classification of social enterprises, proposed criterion of financial soundness level, makes it possible to justify the choice of funding source which affects the process of harmonizing social and business goals.

Many successful examples from foreign practice that deals with the creation and functioning of social enterprises with achievement of significant financial results, great social synergistic effect and attraction of local government and society for cooperation, can be cited.

For example, in the Netherlands, it is common practice to create social enterprises for homeless people, such as workshops for repairing bicycles, making candles, restoring old furniture etc. It is important that the operating expenses of the organization are covered by the local budget.

For the group of people with mental illness and disabilities, a number of social enterprises in the Netherlands such as restaurants, bakeries and printing services provide full inclusion of this segment of the population in social life.

In Canada, for example, there is a social enterprise, collecting and selling second-hand products for the low-income groups of the population. It is important that this enterprise carry out vocational training and employ people with disabilities. Thus, the profits are used to meet social needs. Moreover, the local population is actively involved in collecting and supplying second-hand products.

The Canadian practice of operating courier services is also worth mentioning. The main workers are people with mental illness. There are a number of other social enterprises in various spheres of business that attract various groups from the population of the territorial community.

The interesting model of successful bank financing in social entrepreneurship is the Motivation Clinic (Romania, Bucharest). The Motivation Clinic, opened in 2011, is a social enterprise in the field of rehabilitation for patients with orthopedic (fractures, muscle and back pain), neurological (cerebral palsy, spinal cord injuries, insult, muscular dystrophy), and rheumatic diseases. This is an example of private business with paid services, where the social program provides free care for people with disabilities. Currently, the clinic has 45 paid patients, 12 of whom are disabled and receive 25% discount on special package of services. This number of patients enable two people with disabilities to be served freely for a month. The selection criterion is the cumulative monthly family income (below 120 euros per person). The clinic was opened through loan obtained from one of the Romanian banks under the special program of lending to social enterprises: at 0%, without commissions and collateral.

One more example, funding by EU specialized structures, is the Mestesukar Butiq store (Bucharest, Romania). The shop was created as a small cooperative for the sale of handicrafts, created by national minorities, in particular, by the Romanian population. The profit is spent on social programs. This social enterprise operates within the framework of a large integration project, aimed at restoring traditional crafts. The ultimate goal of the project is the establishment of 80 professional cooperatives in Romania. The program was designed for 5 years (2010–2015) and it was funded by EU structures. The budget is 5 million euros (Diamond, 2015 p 141–155).

In this study, of particular interest is the WNISEF Foundation's Social Funding Program (funded by the US government through the United States Agency for International Development (USAID). It develops mechanism for granting soft loans to social enterprises, with joint implementation by commercial partner banks. The analysis of loan conditions is given in Table 5 (Nair and Saiz-Alvarez, 2019).

**Table 5:** The conditions for granting preferential loans to social enterprises.

The loan amount	From 10,000 to 100,000 US dollars
Credit rate	5–10% per annum (fixed rate for the entire credit period). The rate will be set individually by the credit committee of the program.
Credit terms	Up to 36 months (3 years) for investment credits up to 24 months (2 years) for replenishing working capital
Terms of the credit	100% (all loan amount at a time) or non-revolving line of credit (issuing a credit amount in tranches)
Terms of credit repayment	Standard repayment schedule (% accrued on the balance of debt on the credit body). Annuity (in equal parts) Other (by agreement with the bank)
Targeted use	The purchase of equipment, replenishment of working capital

Since its establishment, WNISEF's cumulative investment has amounted to more than USD $ 185 million for 129 companies, totaling about 27,000 people, and has provided loans to the sum of USD $ 1.8 billion for companies in Ukraine and Moldova.

Our Future Funding of regional social programs is an example of successful implementation of various instruments for social entrepreneurship financing in Russia. This Foundation is a non-profit organization that aimed at qualitative social change by the investment of resources and knowledge in the development of social entrepreneurship as a new phenomenon in the social and economic life of the country. “Our Future” Fund realize tasks with the help of organizing competitions for projects of social entrepreneurs since 2008. Today, the competition has the All-Russian status. Over the entire existence of the All-Russian Social Entrepreneurship Project Contest, the Fund has supported 143 projects from 48 regions of Russia. The total amount of paid loans has amounted to 335,800,000 rubles. Comprehensive support, realized by the Our Future Fund for beginning social entrepreneurs includes: financial support in the form of grants, loans and participation in authorized capital; training and consulting on topical issues of their activities, including business planning, etc (The annual report on the Foundation of Regional Social Programs, 2017).

## 5 Discussion

On the basis of the conducted research, it can be argued that social entrepreneurship is an activity realized with the aim of solving or mitigating the problems in socially unprotected segments of the population, on the conditions of self-support, innovation and financial independence. It is a fundamentally new form of entrepreneurship, with successful combination of social goals and commercial practice. The social entrepreneurship is a symbiosis of charity and business approaches for solving social problems. The social entrepreneurship takes social orientation from charity and from business, it takes the entrepreneurial approach. The activity of social enterprises is based on the principles of socially responsible business, reflecting the socio-economic interests of personnel, society and the operating territory.

The key to the successful development and functioning of social entrepreneurship is the financial and resource support of large business corporations.

Almost in all countries are there assistance of social enterprises from the government, so laws are created, at different levels with financial and legislative conditions for the development of such enterprises. However, in the European countries, unlike the United States, this support is more and exist at all levels of government.

## 6 Conclusion

In our opinion, the investment openness of such enterprises and transparent accounting system should be an important driver for the further development of social entrepreneurship. It will create conditions for the formation of social financial market. The financial resources in social entrepreneurship should be directed towards replicating models that are effective in practice. On the one hand, it may affect the innovativeness of the activities in social enterprises, and on the other hand, it will also contribute to financial stability. In subsequent years, it is expected that government's expenditure in the social sphere will be reduced and accumulated in organizing programs with expected results. Priority will be given to those social enterprises that use effective models of monetization of social influence, which lead to the consolidation of social businesses.

Despite the fact that social enterprises are engaged in business, they actively use government subventions and attract private funds for activities. The government in different countries supports social enterprises in different ways: from government grants to tax breaks and compensation for part of the employees' salaries. The existence of such social enterprises without additional state and public support is impossible. The reason for this is that achievement of economic efficiency and independence, using the occupation of this kind of workers, is almost impossible. The government should clearly identify what social enterprises need and what social tasks they will solve.

The qualitatively new level of development for social entrepreneurship will depend on the establishment of inter-sectoral cooperation by key partners such as: government authorities and local governments (regulatory support, the main mechanism of financial support); business (mentoring, grant support); media (promotion of activities); the educational institutions (social entrepreneurship training course) and public organizations (working directly with the public with the purpose of clarifying the value of key principles of social enterprises).

Each of these players can contribute to the development of social entrepreneurship and investment: the state - by creating a transparent legal framework that is consistent with the reality, by outsourcing social services, by means of guarantee and concession mechanisms for investing in projects of social enterprises; big business by using various forms of social investment and training, and consulting public organizations – by the promotion of activities and the exchange of experience.

## Declarations

### Author contribution statement

Nurkhodzha Akbulaev: Conceived and designed the experiments; Performed the experiments; Analyzed and interpreted the data; Wrote the paper.

Yusif Aliyev: Contributed reagents, materials, analysis tools or data.

Turan Ahmadov: Performed the experiments; Contributed reagents, materials, analysis tools or data.

### Funding statement

This research did not receive any specific grant from funding agencies in the public, commercial, or not-for-profit sectors.

### Competing interest statement

The authors declare no conflict of interest.

### Additional information

No additional information is available for this paper.
